# The role of lifestyle factors on comorbidity of chronic liver disease and cardiometabolic disease in Chinese population: A prospective cohort study

**DOI:** 10.1016/j.lanwpc.2022.100564

**Published:** 2022-08-10

**Authors:** Yuanjie Pang, Yuting Han, Canqing Yu, Christiana Kartsonaki, Yu Guo, Yiping Chen, Ling Yang, Huaidong Du, Wei Hou, Danile Schmidt, Rebecca Stevens, Junshi Chen, Zhengming Chen, Jun Lv, Liming Li

**Affiliations:** a Department of Epidemiology & Biostatistics, School of Public Health, Peking University, 38 Xueyuan Road, Beijing 100191, China; b Peking University Center for Public Health and Epidemic Preparedness & Response, 38 Xueyuan Road, Beijing 100191, China; c Clinical Trial Service Unit & Epidemiological Studies Unit (CTSU), Nuffield Department of Population Health, Big Data Institute Building, Roosevelt Drive, University of Oxford, UK; d Medical Research Council Population Health Research Unit (MRC PHRU) at the University of Oxford, Nuffield Department of Population Health, University of Oxford, UK; e Chinese Academy of Medical Sciences, 9 Dongdan San Tiao, Beijing 100730, China; f Licang Center for Disease Prevention and Control, 20 Yongnian Road, Licang District, Qingdao 266041, China; g National Center for Food Safety Risk Assessment, 37 Guangqu Road, Beijing 100021, China; h Key Laboratory of Molecular Cardiovascular Sciences (Peking University), Ministry of Education, 38 Xueyuan Road, Beijing 100191, China

**Keywords:** Chronic liver disease, Cardiometabolic disease, Comorbidity, Lifestyle, Chinese

## Abstract

**Background:**

Lifestyle factors are associated with chronic liver disease (CLD) and death after CLD diagnosis. However, their associations with pathways of CLD progression have been unclear, particularly transition to cardiometabolic disease (CMD), a major comorbid condition with CLD. We assessed the associations of lifestyle factors with CLD progression.

**Methods:**

The study population involved 486,828 participants of the prospective China Kadoorie Biobank (CKB) aged 30-79 years without a history of cardiovascular disease, diabetes, CLD, or cancer at baseline. Liver-cardiometabolic comorbidity (LCC) was defined as developing CMD subsequently after first CLD (FCLD) in an individual. A multi-state model was used to estimate the associations of high-risk lifestyle factors (smoking, alcohol, physical inactivity, and central adiposity) with CLD progression from healthy to FCLD, subsequently to LCC, and further to death.

**Findings:**

During a median follow-up of 11 years, 5046 participants developed FCLD, 519 developed LCC, and 157 died afterwards. There were positive associations between the number of high-risk lifestyle factors and risks of all transitions. The hazard ratios (95% CIs) per 1-factor increase were 1.30 (1.25-1.35) for transitions from baseline to FCLD, 1.21 (1.09-1.34) for FCLD to LCC, 1.20 (1.17-1.23) for baseline to death, 1.15 (1.09-1.22) for FCLD to death, and 1.17 (1.06-1.31) for LCC to death. For CLD subtypes, lifestyle factors showed different associations with disease-specific transitions even within the same transition stage.

**Interpretation:**

High-risk lifestyle factors played a key role in all disease transition stages from healthy to FCLD, subsequently to LCC, and then to death, with different magnitude of associations.

**Funding:**

Kadoorie Charitable Foundation, Chinese MoST and NSFC.

**Research in context**

**Evidence before this study**

We searched Pubmed from database inception to December 1, 2021, with the terms “lifestyle” AND “liver” AND “cardiometabolic disease”. We included prospective cohort studies in the general population. We excluded studies involving high-risk populations (e.g. obese or diabetic participants) or participants under 18 years of age, animal studies, and review articles.

We identified no studies assessing the associations of lifestyle in relation to cardiometabolic comorbidity among chronic liver disease (CLD) patients. Several small-scale prospective studies of CLD patients examined the associations of individual lifestyle factors with risk of death. These studies showed that smoking was associated with higher risk of death and obesity was associated with lower risk of death among CLD patients. A meta-analysis of including 408,330 European adults showed that a healthy lifestyle (avoidance of smoking, limited alcohol consumption, physical activity, healthy diet, and body weight) was associated with lower risk of liver cancer mortality, but no studies assessed the associations of lifestyle factors with progression after CLD.

**Added value of this study**

The present study is one of the first to examine the associations of lifestyle factors in relation to CLD progression, with special emphasis on cardiometabolic comorbidity. It is important to understand the transitions after CLD diagnosis as well as the role lifestyle factors play in such transitions, in order to inform primary and secondary prevention of CLD. We found that high-risk lifestyle factors played a key role in all disease transition stages from healthy to first CLD (FCLD), to liver-cardiometabolic comorbidity (LCC), and then to death, with different magnitude of associations. Lifestyle factors showed stronger associations with transition from baseline to FCLD than from FCLD to LCC.

**Implications of all the available evidence**

Given the poor prognosis of CLD, our findings highlight the need for clinicians to promote lifestyle interventions among CLD patients in early stage of disease course, in order to prevent transitions to LCC and death.

Alt-text: Unlabelled box

## Introduction

Chronic liver disease (CLD), including alcoholic liver disease (ALD), non-alcoholic fatty liver disease (NAFLD), viral hepatitis, cirrhosis, and liver cancer, is a major burden of disease globally.^1^ According to the Global Burden of Disease project, the total number of deaths globally in 2017 was 0.82 million for liver cancer and 2.2 million for cirrhosis.^2^^,^^3^ Multimorbidity is commonly defined as the presence of 2 or more diseases in an individual.^4^ While “multimorbidity” is often used interchangeably with “comorbidity”, the former refers to the co-occurrences of multiple conditions with no single condition holding priority, and the latter refers to the combined effects of additional conditions in reference to an index chronic condition.^5^

Cardiometabolic disease (CMD), including coronary heart disease (CHD), stroke, and diabetes, is a common comorbid condition among CLD patients.^6^ Cardiovascular disease (CVD) is one of the three most common causes of mortality in NAFLD patients (30%-62% of cases).^7^ The prevalence of comorbid conditions in cirrhosis patients was 12.7% for diabetes, 6.6% for CHD, and 5.5% for cerebrovascular diseases.^8^ Observational studies have reported that CLD patients had higher risk of developing diabetes and CVD.^6^ The associations between CLD and CMD might be due to shared risk factors (e.g. smoking, adiposity, physical inactivity) and reflect underlying aetiology such as insulin resistance and inflammation.^9^, ^10^, ^11^, ^12^

Lifestyle factors, including smoking, alcohol, physical inactivity, and adiposity, are associated with risk of CLD in Western countries and in China.^13^, ^14^, ^15^, ^16^, ^17^ However, it is not clear the impact of lifestyle factors on the progression from CLD to CMD, and further to death. It is also not clear whether such patterns of associations differ by CLD subtypes. It is important to understand the transitions after CLD diagnosis as well as the role lifestyle factors play in such transitions, in order to inform primary and secondary prevention of CLD. Therefore, we aimed to examine (1) the associations of lifestyle factors with transitions from free of CLD, subsequently to liver-cardiometabolic comorbidity (LCC), and further to death and (2) whether the associations differ by CLD subtype.

## Methods

### Study population

Details of the CKB design, survey methods and population characteristics have been described elsewhere.^18^ Briefly, 512,715 participants (210,205 men and 302,510 women) aged 30-79 years were recruited into the study from 10 (5 urban and 5 rural) geographically defined localities in China during 2004-2008. The study areas were selected to provide diversity in risk exposure and disease patterns, while taking into account population stability, quality of mortality and morbidity registries, capacity, and long-term commitment within the areas. Prior international, national and regional ethical approvals were obtained, and all participants provided written informed consent. At local study assessment clinics, participants completed an interviewer-administered laptop-based questionnaire on socio-demographic characteristics, smoking, alcohol consumption, diet, physical activity, personal and family medical history and current medication. A range of physical measurements were recorded by trained technicians, including height, weight, hip and waist circumference, bio-impedance, lung function, blood pressure and heart rate, using calibrated instruments with standard protocols.

### Data collection on lifestyle risk factors

Individuals were classified by smoking status as never, occasional, former regular, or current regular smokers. Never smokers were defined as those who reported not smoking at baseline and had smoked <100 cigarettes (or equivalent) in their lifetime. Former regular smokers were defined as those who had smoked ≥100 cigarettes (or equivalent) but had quit smoking by choice for ≥6 months before baseline. Occasional smokers were defined as those who did not meet the criteria for never smokers, and who had not stopped smoking completely for ≥6 months before baseline. Current regular smokers were defined as those who reported having ever smoked ≥1 cigarette (or equivalent) daily for at least 6 months. Approximately 50% of former regular smokers stopped smoking because of physical illness that they already had, and they were still considered as current regular smokers in the main analyses.

The information on alcohol drinking included whether the participant had drunk alcohol regularly (i.e. drank at least once a week on a regular basis) during the past year, and if so, the age at which drinking began, the type (beer, wine or spirits) and the amount consumed on a typical drinking day. Abstainers were defined as those who had never or almost never drunk alcohol in the past year and had not drunk weekly in the past. Occasional drinkers were defined as those who in the past year had drunk alcohol occasionally, during certain seasons, or monthly but less than weekly, and had not drunk weekly in the past. Reduced-intake drinkers were those who in the past year had drunk alcohol occasionally, during certain seasons, or monthly but less than weekly, but had drunk weekly in the past. Ex-weekly drinkers were those who had drunk weekly in the past but had never or almost never drunk alcohol in the past year. Weekly drinkers were those who usually drank at least once a week during the past year. Weekly alcohol consumption was collected in weekly drinkers including beer, wine, and spirits and was calculated from frequency per week and amount per day.

Participants were asked about their usual type and duration of activities related to work, commuting, household chores, and leisure-time exercise during the past year. To quantify the amount of physical activity, metabolic equivalent of tasks (METs) from the 2011 update of a major compendium of physical activities were used.^19^ The MET value for a particular type of physical activity represents the ratio of the energy expended per kilogram of body weight per hour during that activity to that expended when sitting quietly. The number of hours spent per day participating in each activity was multiplied by the MET score for that activity, and the daily amount of total physical activity was obtained by summing the MET-hours for activities related to occupation and non-occupational (i.e. commuting, housework, and non-sedentary leisure-time activities) activities.

Standing height was measured using a stadiometer. Weight was measured using a body composition analyzer (TANITA-TBF-300GS; Tanita Corporation), with subtraction of weight of clothing according to season (ranging from 0.5 kg in summer to 2.0-2.5 kg in winter). Waist circumference (WC) and hip circumference (HC) were measured using a soft non-stretchable tape, with HC measured at the maximum circumference around the buttocks. BMI at baseline was calculated as the measured weight in kilograms divided by the square of the measured height in meters. Waist-to-hip ratio (WHR) is the ratio of WC to HC.

### Follow-up for and ascertainment of disease cases

The vital status of each participant was determined periodically through China CDC's Disease Surveillance Points (DSP) system, supplemented by regular checks against local residential and administrative records and by annual active confirmation through street committees or village administrators.^20^ In addition, information about major diseases and any episodes of hospitalization was collected through linkages, via each participant's unique national identification number, with disease registries (for cancer, ischemic heart disease, stroke, and diabetes) and national health insurance claims databases. All disease events were coded using International Classification of Diseases, 10th Revision (ICD-10) by trained DSP staff (for death) or medical professionals (for hospitalised events) who were blinded to baseline information. The present study included incident liver diseases and liver cancer from enrolment until December 31, 2017 (a median of 10 years), by which time a total of 42,921 (8%) participants had died and 5,276 (1%) were lost to follow-up. The classification and distribution of liver diseases by data source are shown in Supplementary Table 1 (NAFLD 100% by medical records). Liver cancer was defined by ICD-10 code C22. Secondary cancer was coded as C78.7. CMD was defined as having one of four individual diseases of ischemic heart disease (IHD, ICD-10: I20-I25), ischemic stroke (IS, I63), haemorrhagic stroke (HS, I61), and type 2 diabetes (T2D, E11 and E14). LCC was defined as developing CMD subsequently after first CLD in an individual.

### Definition of lifestyle risk factors

We selected smoking, alcohol, physical activity, and central adiposity to construct a combined high-risk lifestyle score. This is because these lifestyle factors have been shown to be associated with risk of CLD in the Chinese population.^16^^,^^17^^,^^21^ To investigate the combined effects of high-risk lifestyle, we grouped each participant into 1 of 4 categories according to the number of high-risk lifestyle factors (0-3), including smoking (current or former regular smokers), alcohol (weekly alcohol consumption ≥210 g, ex-regular or reduced-intake drinkers), physical inactivity (total physical activity <17.47 MET-h/day [the top 50%]), and central obesity (WC≥90 cm [men] or 80 cm [women]). The cut-off points were selected for each lifestyle factor based on *a priori* knowledge of the risk factors for CLD and are considered achievable at the population level.^22^^,^^23^

### Statistical analysis

**Figure 1:**
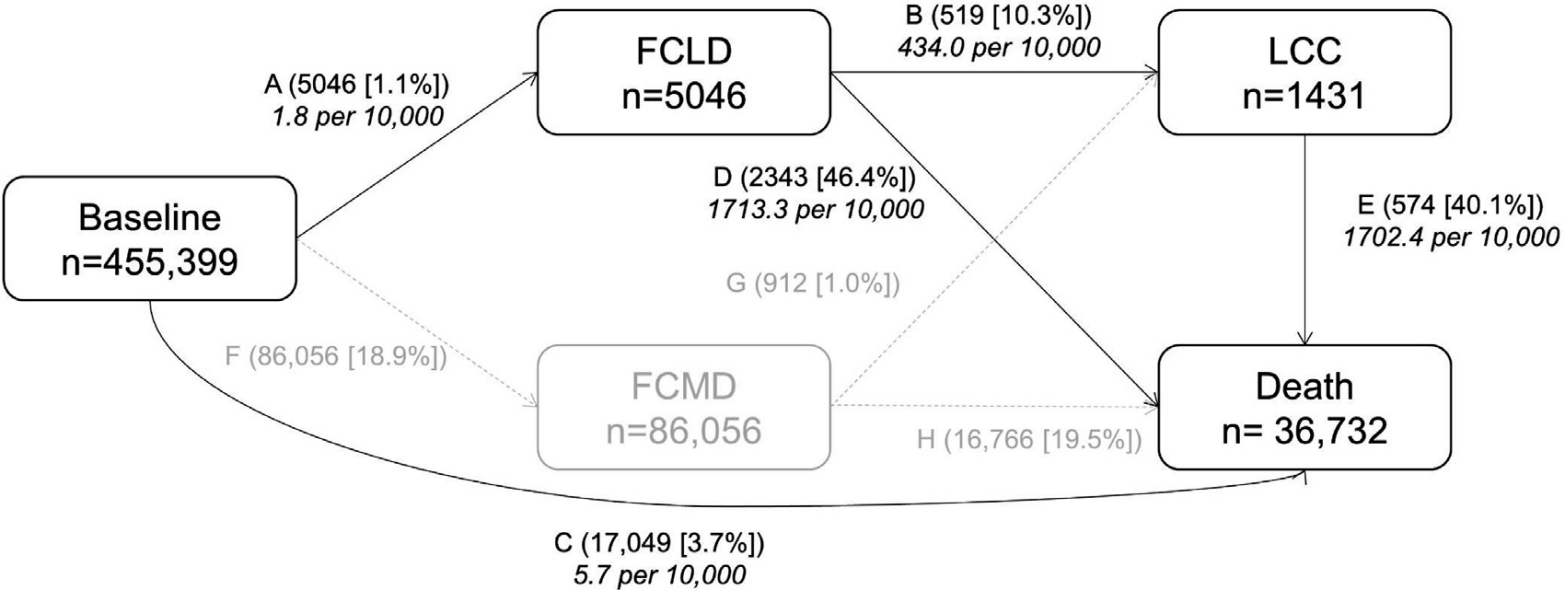
**Numbers (percentages) of participants from baseline to first chronic liver disease (FCLD), liver-cardiometabolic comorbidity (LCC), and death**. CMD includes coronary heart disease, stroke, and type 2 diabetes. LCC is defined as developing CMD subsequently after FCLD in an individual. In path C, baseline healthy to death excluded death from CLD and CMD. Stage-specific number of events is reported in boxes, and transition-specific number of events and proportions are reported on arrows. Incidence rate (per 10,000) is reported in italic text. Abbreviations: FCLD, first chronic liver disease; LCC, liver-cardiometabolic comorbidity.

We used a multi-state model to assess the role of both individual and combined lifestyle factors in the temporal disease progression from free of CLD and CMD to FCLD, LCC, and death. The multi-state model is an extension of the competing risk model and it is useful to explore how certain factors influence different phases of a process.^24^ Detials of the multi-state model is presented in eMethods in the supplementary materials. Eight transition stages were constructed based on the natural history of CLD (Figure 1): (i) baseline healthy to FCLD; (ii) baseline healthy to FCMD; (iii) FCLD to LCC; (iv) FCMD to LCC; (v) baseline healthy to death from a disease other than CLD and CMD; (vi) FCLD to death from any causes; (vii) FCMD to death from any causes; and (viii) LCC to death from any causes. Because the focus is the pathway of CLD progression, we only reported the associations of lifestyle facotrs with transition stages on five pathways (i, iii, v, vi, and viii) (Figure 1). For participants who entered different states on the same date (*n*=6730), we calculated the entering date of the theoretically prior state as the entering date of the latter state minus 0.5 day. For example, for participants who died of FCLD, the date of FCLD occurrence equals the date of death minus 0.5 day. Participants who were diagnosed with at least two CLDs on the same date were excluded because the temporal sequence of disease occurrences could not be ascertained.

We also used the multi-state model with the same setting to analyse the effects of lifestyle factors on transition patterns for subtypes of CLD. FCLDs were divided into four individual diseases (i.e. NAFLD, viral hepatitis, cirrhosis, and liver cancer). ALD was not included as a separate outcome because of the small number of events (*n=*240). In this pattern, CLD was each redefined as having one of the four individual outcomes.

In the analysis of individual lifestyle factors, the multi-state models adjusted for age at baseline, sex, study area (10 regions), education (4 groups: no formal school, primary school, middle/high school, or college/university), BMI, HBsAg, and self-rated health, with additional adjustment for the other lifestyle factors. Time since birth was used as the underlying time scale and participants entered the study at their baseline age. In combined analyses, high-risk lifestyle factors were modelled as a categorical variable (0 to 4 points) and as an ordinal variable (per 1-point increase in number; linear trend). The same variables were adjusted for as in the analysis of individual risk factors, and each lifestyle factor was weighted equally. Adjusted HRs were reported for individuals with 2, 3, and 4 healthy lifestyle factors compared with those with all 0-1 healthy lifestyle factors.

Several sensitivity analyses for the multi-state analyses were conducted: (i) calculating the entering date of the prior state using different time intervals (1, 3, and 5 days) for participants who entered different states on the same day; (ii) excluding participants who entered different states on the same date; (iii) additionally adjusting for hypertension, usage of blood pressure medicine, and statin at baseline; (iv) including participants who had previously diagnosed CLD, CHD, stroke, or diabetes, and assigning them to FCLD, FCMD or LCC state according to their disease status at baseline; (v) excluding the events occurring in the first 2 years of follow-up; (vi) calculating the entering date of the theoretically prior state as half of the entering date of the latter; (vii) examining the associations of lifestyle risk factors with progression from FCLD to LCDD by CMD subtypes.

The multi-state model was performed using “mstate” package of R (version 3.6.3). Two-tailed *p*-value < 0.05 indicated statistical significance.

### Role of the funding source

The funders had no role in the study design, data collection, data analysis and interpretation, writing of the report, or the decision to submit the article for publication.

## Results

### Descriptions of transitions

Of the 455,399 participants included, mean (SD) age was 52 (10.9) years, and 59.2% were women. Lifestyle factors were collected at study baseline. The mean (SD) BMI was 23.8 (3.4) kg/m^2^, and 5.9% had diabetes at baseline. During 10 years of follow-up, the number of incident events were 5046 for FCLD. Of all FCLD patients, 519 (transition B) developed CMD and 2343 (transition D) died from any causes afterward, with an incidence rate of 434.0 and 1713.3 per 10,000 persons; 17,049 (transition C) died without developing CLD, with an incidence rate of 5.7 per 10,000 persons (Figure 1). 300 participants developed at least two CLDs on the same date, accounting for 4.9% of all FCLD. The primary cause of death in transition C, D, and E was cancer (ICD10: C00-C97), accounting for 50.9%, 96.4%, and 83.2% of deaths, respectively. Liver cancer accounted for 57.7% and 40.2% of total deaths in transition D and E, respectively. Participants who died with LCC had higher levels of systolic blood pressure (SBP), while participants who died with FCLD had the lowest levels of adiposity (Supplementary Table 2). Participants who died with LCC were more likely to have 3-4 high-risk lifestyle risk factors.

### Individual lifestyle factors and risk of multimorbidity of CLD and CMD

Smoking was positively associated with FCLD, but not with transition from CLD to LCC (Table 1). Smoking was also positively associated with death without experiencing FCLD as well as transitions from FCLD to death and from LCC to death. Alcohol was positively associated with FCLD, transition from FCLD to LCC, and death without experiencing CLD, but not associated with other transitions. Physical activity was positively associated with death without experiencing CLD and transition from FCLD to death, but was not associated with other transitions. Central adiposity was positively associated with FCLD and transition from CLD to LCC. In contrast, central adiposity was inversely associated with death without experiencing CLD as well as transitions from FCLD to death and from LCC to death.

**Table 1:** Hazard ratios by individual healthy lifestyle factors by CLD subtypes.

		HR (95% CI)			
	No. events	Smoking	Alcohol	Physical inactivity	Central adiposity
**Baseline → FCLD**		**1.51 (1.42, 1.61)**	**1.64 (1.50, 1.79)**	**0.95 (0.89, 1.02)**	**1.09 (1.02, 1.16)**
Baseline → NAFLD	743	1.11 (0.93, 1.31)	1.84 (1.45, 2.34)	0.78 (0.65, 0.92)	3.16 (2.72, 3.67)
Baseline → Viral hepatitis	1638	1.39 (1.25, 1.55)	1.58 (1.36, 1.84)	0.93 (0.82, 1.05)	0.94 (0.84, 1.05)
Baseline → Cirrhosis	1237	1.50 (1.33, 1.69)	0.93 (0.75, 1.14)	1.00 (0.87, 1.15)	0.96 (0.84, 1.08)
Baseline → Liver cancer	1999	1.95 (1.77, 2.14)	1.52 (1.34, 1.74)	1.05 (0.93, 1.18)	0.77 (0.69, 0.85)
**FCLD → LCC**		**1.08 (0.89, 1.30)**	**1.38 (1.08, 1.76)**	**1.15 (0.93, 1.43)**	**1.32 (1.11, 1.57)**
NAFLD → LCC	182	0.79 (0.55, 1.12)	1.43 (0.88, 2.33)	1.35 (0.94, 1.94)	0.99 (0.72, 1.34)
Viral hepatitis → LCC	178	1.06 (0.75, 1.50)	1.84 (1.21, 2.81)	1.19 (0.79, 1.81)	0.99 (0.70, 1.39)
Cirrhosis → LCC	135	1.74 (1.21, 2.50)	1.06 (0.57, 1.97)	0.96 (0.63, 1.48)	1.70 (1.19, 2.43)
Liver cancer → LCC	86	1.02 (0.63, 1.66)	1.30 (0.69, 2.44)	1.51 (0.74, 3.10)	1.56 (0.97, 2.49)
**Baseline → Death**	15983	**1.68 (1.62, 1.74)**	**1.30 (1.23, 1.36)**	**1.07 (1.02, 1.12)**	**0.73 (0.70, 0.76)**
**FCLD → Death**		**1.62 (1.48, 1.77)**	**1.03 (0.91, 1.16)**	**1.27 (1.13, 1.42)**	**0.64 (0.58, 0.71)**
NAFLD → Death	25	1.66 (0.65, 4.26)	2.39 (0.90, 6.34)	1.72 (0.57, 5.17)	0.65 (0.28, 1.51)
Viral hepatitis → Death	810	1.60 (1.29, 1.98)	1.35 (1.03, 1.77)	0.94 (0.74, 1.19)	1.09 (0.88, 1.36)
Cirrhosis → Death	391	1.76 (1.42, 2.17)	1.05 (0.74, 1.48)	1.20 (0.92, 1.57)	0.81 (0.64, 1.03)
Liver cancer → Death	1659	1.26 (1.13, 1.41)	0.84 (0.73, 0.98)	1.06 (0.93, 1.21)	0.91 (0.81, 1.03)
**LCC → Death**	645	**1.68 (1.42, 1.99)**	**1.20 (0.96, 1.51)**	**1.18 (0.91, 1.52)**	**0.72 (0.61, 0.84)**

Abbreviations: FCLD, first chronic liver disease; FCMD, first cardiometabolic disease; LCC, liver-cardiometabolic comorbidity.

The model was adjusted for age at baseline, sex, study area, education, BMI, HBsAg, and self-rated health, with additional adjustment for the other lifestyle factors.

### Combined lifestyle factors and risk of multimorbidity of CLD and CMD

**Figure 2:**
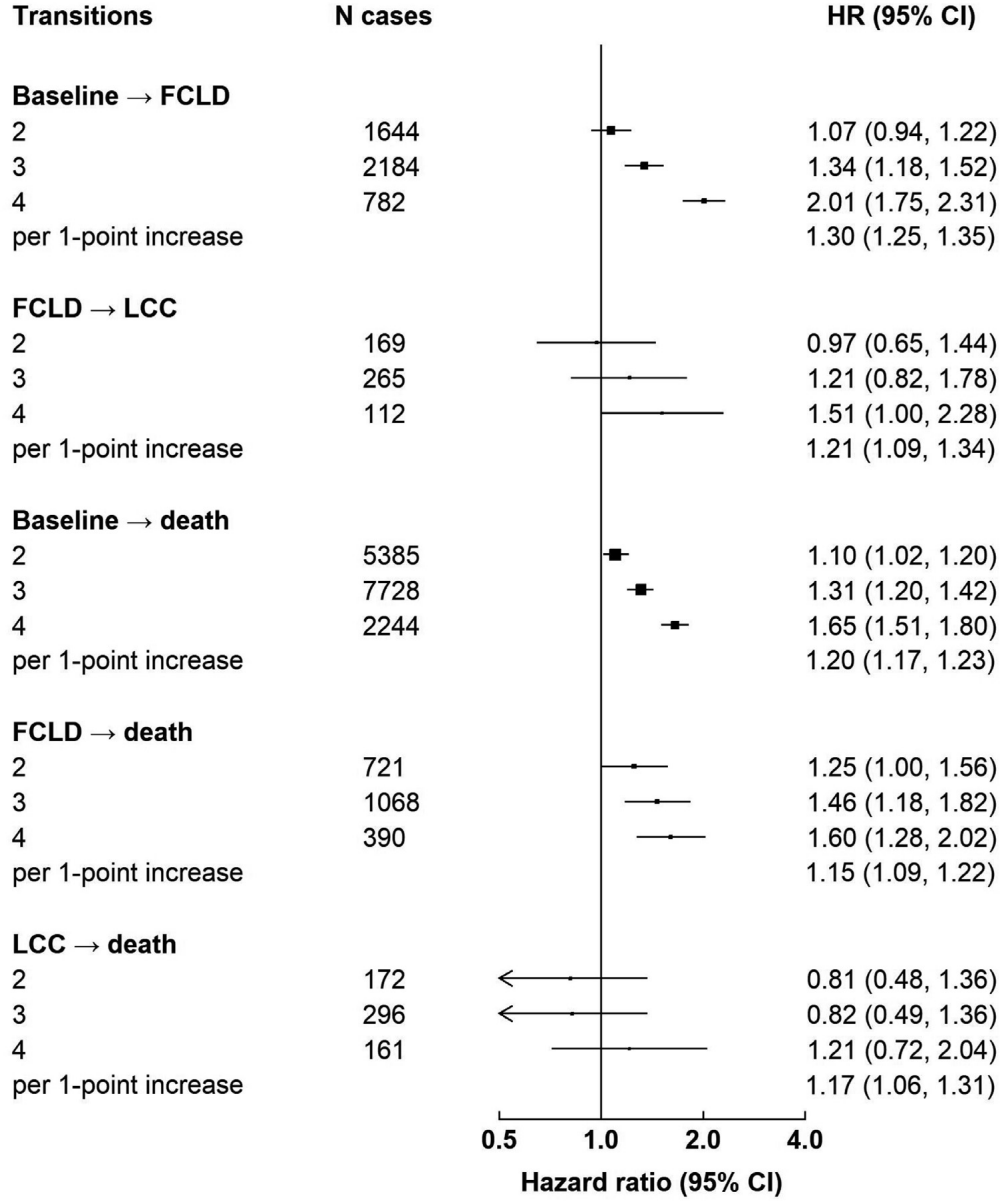
**Hazard ratios by number of high-risk lifestyle factors**. Boxes represent the hazard ratios (HRs) of each transition associated with the number of high-risk lifestyle factors, with the size of the box inversely proportional to the variance of the logHR. The model was adjusted for age at baseline, sex, study area, education, BMI, HBsAg, and self-rated health. Abbreviations: FCLD, first chronic liver disease; FCMD, first cardiometabolic disease; LCC, liver-cardiometabolic comorbidity.

Despite the different magnitude of associations for individual lifestyle factors, there were positive associations between the number of combined lifestyle factors and risks of all transitions (Figure 2). Heterogeneity test showed that the associations of combined lifestyle factors with all transitions differed (*p*-value for heterogeneity 0.003, **eMethods** in supplementary materials). There were stronger associations for transitions from baseline healthy to FCLD than from FCLD to LCC, but the difference was non-significant (*p*-value for heterogeneity 0.21). The associations for mortality outcomes (i.e. baseline to death, CLD to death, and CMD to death) were generally weaker than those for FCLD, with the weakest associations observed for transition from LCC to death. The adjusted HRs per 1-factor increase were 1.30 (1.25-1.35) from baseline healthy to FCLD, 1.21 (1.09-1.34) from FCLD to LCC, 1.20 (1.17-1.23) for baseline healthy to death from a disease other than CLD, 1.15 (1.09-1.22) for FCLD to death from any causes, and 1.17 (1.06-1.31) for LCC to death from any causes, respectively.

When modelling lifestyle factor as a categorical variable, there was a graded increase between the number of lifestyle factors and risks of almost all transitions. Nonetheless, the associations of combined lifestyle factors with transitions from LCC to death when using categorical variables were non-significant possibly because of the small number of cases.

### Lifestyle factors and risk of multimorbidity of CLD by subtypes

The associations of combined lifestyle factors with transitions tended to differ by CLD subtypes (Supplementary Table 3). For baseline to FCLD, the strongest association was observed for NAFLD and the weakest for cirrhosis. For FCLD to LCC, there were positive associations for viral hepatitis, cirrhosis, and liver cancer, but no clear associations for NAFLD. For FCLD to death, there were positive associations for NAFLD, viral hepatitis, and cirrhosis, but no clear associations for liver cancer. Similarly, the associations of individual lifestyle factors with transitions also differed by subtypes of CLD (Table 1).

### Sensitivity analyses

In sensitivity analyses, the associations of lifestyle risk factors with CLD progression were similar to those in the main analyses (Supplementary Table 4). The associations slightly attenuated towards the null when excluding cases in the first two years of follow-up, particularly for physical activity. The associations of lifestyle risk factors with progression from FCLD to LCC did not differ by CMD subtypes (*p*-value for heterogeneity 0.18, Supplementary Table 5).

## Discussion

In this prospective study of 0.5 million Chinese adults, we found that four high-risk lifestyle factors played a key role in all disease transition stages from healthy to FCLD, to LCC, and then to death, with different magnitude of associations. Lifestyle factors showed stronger associations with transition from baseline to FCLD than from FCLD to LCC. The associations for mortality outcomes were generally weaker than those for from baseline to FCLD, with the weakest associations observed for transition from LCC to death. When examining CLD subtypes, lifestyle factors showed different associations with disease-specific transitions even within the same transition stage.

Our findings for incident FCLD and transitions to death after FCLD were generally consistent with previous prospective studies focusing on each transition stage separately. Previous prospective studies in Western countries and in China have shown that individual lifestyle factors, including smoking, alcohol, physical inactivity, and adiposity, are risk factors for liver cancer.^25^^,^^26^ A recent meta-analysis involving 408,330 European adults reported that a healthy lifestyle, consisting of avoidance of smoking, limited alcohol consumption, physical activity, healthy diet, and body weight, was associated with lower risk of liver cancer mortality (HR 0.68 [0.48, 0.97] comparing individuals with the most versus the least healthy lifestyles). Likewise, a recent report in CKB showed that a favourable lifestyle including non-smoking, non-drinking, median or higher level of physical activity, a healthy diet, and a low WHR, was associated with lower risk of liver cancer (HR 0.57 [0.47, 0.68] comparing individuals with 4-5 vs 0-1 healthy lifestyle factors). Among CLD patients, previous studies conducted in Western populations showed that smoking and physical inactivity was each associated with disease progression including death.^27^^,^^28^ However, there were no studies on other lifestyle factors in relation to CLD progression.

Compared with incident events of FCLD, lifestyle factors showed weaker associations for transitions to death.^29^^,^^30^ This finding is consistent with previous observational studies on lifestyle factors and multimorbidity of CMD. For example, a previous report in CKB showed that the adjusted HRs per 1-factor increase were 1.20 (1.19-1.21) for baseline to FCMD, 1.14 (1.11-1.16) for FCMD to cardio-metabolic multimorbidity (CMM), 1.12 (1.10-1.15) for FCMD to death, and 1.10 (1.06-1.15) for CMM to death.^29^ Although adopting a healthy lifestyle is beneficial for secondary prevention, greater benefits will be achieved by promoting lifestyle interventions before developing the first incident events.

For transitions to mortality outcomes, there were inverse associations of central adiposity with all transition stages (baseline to death, CLD to death, and LCC to death). This “obesity paradox” agrees with previous observational studies showing that adiposity is associated with lower risk of death after developing major chronic diseases.^31^ Indeed, the inverse associations between adiposity and transitions to death are consistent with previous observational studies on multimorbidity that also used multi-state models.^29^^,^^30^ As a result, the associations of lifestyle factors with transitions to death may be somewhat “diluted” by inclusion of central adiposity.

We observed different associations of lifestyle factors with disease-specific transitions for CLD subtypes even within the same transition stage. For FCLD to LCC, there were positive associations for viral hepatitis, cirrhosis, and liver cancer, but no clear associations for NAFLD. Previous studies suggested bi-directional associations between NAFLD and CMD and insulin resistance plays a key role.^6^^,^^9^ However, when diagnosed, NAFLD patients may have already developed subclinical CMD, and therefore, lifestyle factors may not play a role.^32^ Therefore, the “transition” from NAFLD to LCC might reflect disease ascertainment rather than natural history. For FCLD to death, there were positive associations for NAFLD, viral hepatitis, and cirrhosis, but no clear associations for liver cancer. This suggests that lifestyle modifications may not be as effective for secondary prevention of liver cancer. Indeed, the latency period of liver cancer is long and can take decades.^33^ Therefore, lifestyle modification may not be as effective in pre-clinical periods. In addition, reverse causality is likely to affect the associations between lifestyle factors and transition to death. However, reverse causality should be minimised in our study because lifestyle factors were collected during the baseline survey, ∼10 years before the development of liver cancer.

The strengths of the CKB included a prospective design, a large and diverse study population, the validity of CLD diagnosis, and detailed adjustment for risk factors for CLD and CMD. In particular, we ascertained CLD through linkages to hospital records in addition to death and cancer registries, which allowed us to examine different CLD subtypes. We also used the multi-stage models to assess the associations of lifestyle factors on each transition in the progression trajectory of LCC. Our study also had several limitations. First, some participants were diagnosed with CLD and CMD simultaneously in one admission and had the same diagnosis date for both diseases. We assigned an interval of 0.5 day to differentiate the onset date of CLD or CMD, which might by somewhat arbitrary. However, we used different time intervals and dropped participants with different diagnoses on the same date. None of these sensitivity analyses altered the results materially. Second, CLDs that were asymptomatic may be diagnosed as a comorbid condition with CMD (e.g. NAFLD, viral hepatitis), which would bias the associations of lifestyle factors and transitions of CLD. However, we found similar associations when dropping participants with different diagnoses on the same date or when excluding CMD cases that developed 30 days before or after CLD diagnosis. Third, we used lifestyle factors collected at baseline and did not consider possible changes during follow-up. However, previous reports in CKB showed that, the majority of participants had not changed their risk level of lifestyle factors during a median interval of around 8 years.^29^ The use of lifestyle factors assessed at baseline may help avoid reverse causation resulted from lifestyle changes after disease onset. Fourth, smoking is a key risk factor for lung cancer, which accounted for 13.7%, 2.7%, and 3.7% of total deaths in transition C, D, and E, but lung cancer has no causal relationship with LCC. Including lung cancer death may bias the association of smoking with transition from LCC to death. However, given the strong association between smoking with liver cancer (accounting for 40.2% of total death in LCC) and the small proportion of lung cancer death, the effect should be minimal. Fifth, liver cancer included both hepatocellular carcinoma and cholangiocarcinoma, with the latter not related to LCC. However, the small proportion of cholangiocarcinoma (C22.1, 14.3%) might have diluted the association for liver cancer. Lastly, residual confounding due to unmeasured or unknown factors (e.g. infections, lipids, inflammation) may still exist.

In conclusion, high-risk lifestyle factors played a key role in the incidence of FCLD as well as transitions from FCLD to LCC, from baseline healthy to death, from FCLD to death, and from LCC to death, but they played a more modest role in transition from LCC to death. High-risk lifestyle factors showed different associations with disease-specific transitions by CLD subtypes even within the same transition stage. Given the poor prognosis of CLD, our findings highlight the need for clinicians to promote lifestyle interventions among CLD patients in early stage of disease course, in order to prevent transitions to LCC and death.

## Contributors

YP and JL conceived and designed the paper. LL, ZC, and JC, as the members of CKB steering committee, designed and supervised the conduct of the whole study, obtained funding. DS and RS acquired the data. YP and YH analysed the data. YP drafted the manuscript. YP, YH, CY, CK, and JL contributed to the interpretation of the results. All authors critically reviewed and revised the manuscript for important intellectual content. All authors reviewed and approved the final manuscript. JL is the guarantor. All authors had access to all data and responsibility for the decision to submit for publication.

## Data sharing statement

The access policy and procedures are available at www.ckbiobank.org.

## Declaration of interests

We declare that we have no conflict of interest.

## Appendix Supplementary materials

Image, application 1
