# Supplementary material for: The role of lifestyle factors on comorbidity of chronic liver disease and cardiometabolic disease in Chinese population: A prospective cohort study
Source: Lancet Reg Health West Pac. 2022 Aug 10;28:100564. doi: 10.1016/j.lanwpc.2022.100564 (PMC9386629; doi:10.1016/j.lanwpc.2022.100564)
Supplement: Supplementary file 1 [file mmc1.docx]

**Supplementary Material**

**The role of lifestyle factors on comorbidity of chronic liver disease and cardiometabolic disease in Chinese population: a prospective cohort study**

**The Lancet Regional Health - Western Pacific**

Yuanjie Pang, DPhil^1^, Yuting Han, PhD^1^, Canqing Yu, PhD^1,2^, Christiana Kartsonaki, DPhil^3,4^, Yu Guo, MSc^5^, Yiping Chen, DPhil^3,4^, Ling Yang, PhD^3,4^, Huaidong Du, PhD^3,4^, Wei Hou, MS^6^, Danile Schmidt, MS^3^, Rebecca Stevens, MS^3^, Junshi Chen, MD^7^, Zhengming Chen, DPhil^3,4^, Jun Lv, PhD^1,2,8^, Liming Li, MD^1,2^

1. Department of Epidemiology & Biostatistics, School of Public Health, Peking University, 38 Xueyuan Road, Beijing 100191, China
2. Peking University Center for Public Health and Epidemic Preparedness & Response, 38 Xueyuan Road, Beijing 100191, China
3. Clinical Trial Service Unit & Epidemiological Studies Unit (CTSU), Nuffield Department of Population Health, Big Data Institute Building, Roosevelt Drive, University of Oxford, UK
4. Medical Research Council Population Health Research Unit (MRC PHRU) at the University of Oxford, Nuffield Department of Population Health, University of Oxford, UK
5. Chinese Academy of Medical Sciences, 9 Dongdan San Tiao, Beijing 100730, China
6. Licang Center for Disease Prevention and Control, 20 Yongnian Road, Licang District, Qingdao 266041, China
7. National Center for Food Safety Risk Assessment, 37 Guangqu Road, Beijing 100021, China
8. Key Laboratory of Molecular Cardiovascular Sciences (Peking University), Ministry of Education, 38 Xueyuan Road, Beijing 100191, China

**Address for correspondence:**

| Prof. Jun Lv |
| --- |
| Department of Epidemiology and Biostatistics |
| School of Public Health |
| Peking University |
| 38 Xueyuan Road |
| Beijing, 100191, China |
| Tel: 86-010-8280-1528 |
| Fax: 86-010-82801530 |
| lvjun@bjmu.edu.cn |

**Table of content**

[eMethods 3](#_Toc28043)

[Supplementary Table 1. Classification and distribution of liver diseases by ICD-10 codes in CKB 6](#_Toc22419)

[Supplementary Table 2. Baseline characteristics of participants by incident disease status during follow-up 7](#_Toc32667)

[Supplementary Table 3. Hazard ratios by number of healthy lifestyle factors by CLD subtypes 9](#_Toc24468)

[Supplementary Table 4. Sensitivity analyses 10](#_Toc22570)

[Supplementary Table 5. Hazard ratios by number of healthy lifestyle factors by CMD subtypes 13](#_Toc731)

## eMethods

*Multi-state model*

A multi-state model is used for time-to-event data in which all individuals start in one (or more) starting state(s) (e.g. CLD) and eventually end up in one (or more) absorbing or final state(s) (e.g. death). One or more intermediate states can happen in between. Competing risks models are a sub-category of multi-state models. They have one starting state, at least two absorbing states and no intermediate states (**Figure A**). **Figure B** shows an example of a simple multi-state model.

**Figure A. A competing risks model for patients with CLD**


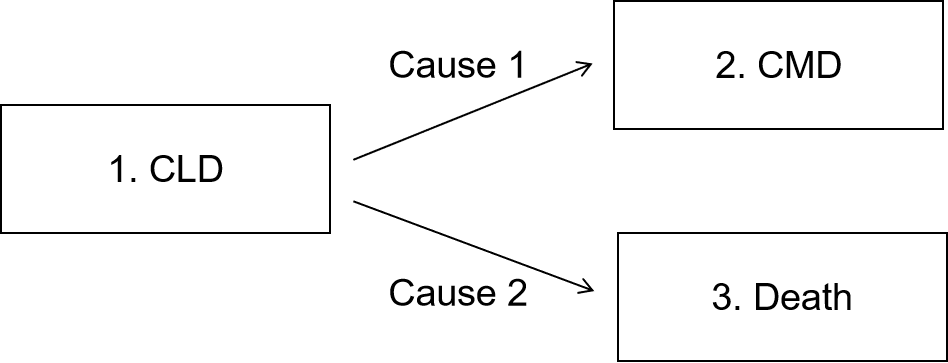


**Figure B. A multi-state model for patients with CLD**


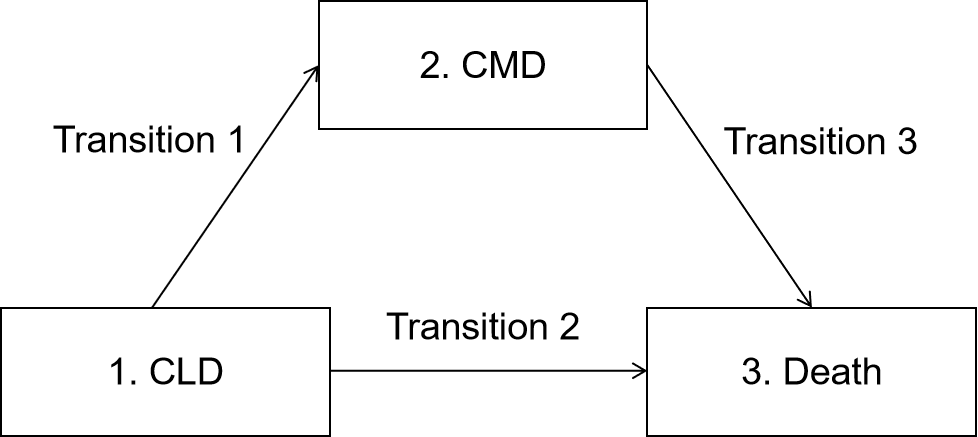


The multi-state model and Cox proportional hazard model have different hazard functions (**definition 1-2**).

In multi-state model, the hazard function, or the instantaneous rate of a transition from state g into state h at time t, defined as (**definition 1**):

$$\alpha_{gh}(t)=\lim_{\Delta t\to0} \frac{P(X(t+\Delta t) = h | X(t) = g)}{\Delta t}$$

In Cox proportional hazard model, the hazard function, or instantaneous rate of occurrence of the event, defined as (**definition 2**):

$$\lambda(t)=\lim_{\Delta t\to0} \frac{P(t\leq T<t+\Delta t | T\geq t)}{\Delta t}$$

*Test for heterogeneity*

The R function (“heterogeneity”) below was used to calculate *p*-value for heterogeneity.

heterogeneity <- function(beta, se) {

# beta must be a vector of estimates

# se must be a vector of their standard errors

# df denotes degrees of freedom

df <- length(beta) - 1

# expected_beta: inverse variance weighted average of betas

expected_beta <- sum(beta / se^2) / sum(1 / se^2)

heterogeneity_test_statistic <- sum(((beta - expected_beta) / se)^2)

p <- pchisq(heterogeneity_test_statistic, df = df, lower = FALSE)

return(list("test statistic" = heterogeneity_test_statistic, "p" = p))

}

# example: p-value for heterogeneity by sex (beta coefficient and SE in men: beta1, se1; women: beta2, se2)

heterogeneity(beta=c(beta1, beta2), se=c(se1, se2))

## Supplementary Table 1. Classification and distribution of liver diseases by ICD-10 codes in CKB

| **Liver disease** | **ICD-10 code** | **No. of cases** | | | |
| --- | --- | --- | --- | --- | --- |
|  |  | **Total** | **Health insurance** | **Death registry** | **Disease registry** |
| Liver cancer | C22 | 2397 | 1164 | 1706 | 704 |
| Cirrhosis | K74 | 1961 | 1516 | 671 | 12 |
| NAFLD | K76.0 | 1216 | 1216 | 0 | 0 |
| ALD | K70 | 265 | 196 | 77 | 1 |
| Viral hepatitis | B18-B19 | 1415 | 1084 | 357 | 2 |

Abbreviations: ALD, alcoholic fatty liver disease; NAFLD, non-alcoholic fatty liver disease.

## Supplementary Table 2. Baseline characteristics of participants by incident disease status during follow-up

|  | **LCC free** | **FCLD survivor** | **FCMD survivor** | **Dead without LCC** | **Dead with FCLD** | **Dead with FCMD** | **Dead with LCC** |
| --- | --- | --- | --- | --- | --- | --- | --- |
| **Variable** | (n=345,181) | (n=2039) | (n=84,814) | (n=15,983) | (n=973) | (n=6141) | (n=252) |
| Age (SD), year | 49.7 (10.1) | 49.7 (9.3) | 55.8 (9.9) | 60.3 (10.5) | 58.8 (10.6) | 63.0 (9.8) | 62.5 (9.3) |
| Female, % | 59.9 | 53.7 | 60.9 | 41.4 | 39.5 | 43.7 | 34.1 |
| **Socioeconomic and lifestyle factors** |  |  |  |  |  |  |  |
| Urban region, % | 42.5 | 33.8 | 44.5 | 34.2 | 32.6 | 27.4 | 35.6 |
| ≥9 years of education, % | 20.9 | 21.3 | 20.0 | 16.6 | 13.8 | 16.7 | 10.9 |
| Household income ≥35 000 RMB/year, % | 18.0 | 17.4 | 18.1 | 14.4 | 11.9 | 12.6 | 9.0 |
| Ever regular smoking, % |  |  |  |  |  |  |  |
| Male | 62.2 | 65.7 | 63.3 | 68.3 | 64.2 | 68.1 | 54.0 |
| Female | 2.2 | 2.6 | 2.3 | 3.0 | 2.0 | 4.2 | 2.8 |
| Weekly drinking, % |  |  |  |  |  |  |  |
| Male | 34.2 | 38.5 | 34.1 | 38.0 | 38.3 | 37.3 | 32.4 |
| Female | 2.2 | 2.3 | 2.0 | 2.1 | 3.7 | 4.3 | 0.0 |
| Total physical activity (SD), MET-h/day | 22.0 (13.9) | 21.6 (14.2) | 21.4 (12.9) | 20.8 (13.3) | 19.2 (13.0) | 19.6 (11.9) | 14.6 (12.4) |
| Sedentary leisure time (SD), h/day | 3.0 (1.5) | 3.1 (1.6) | 3.0 (1.5) | 3.0 (1.7) | 2.7 (1.6) | 3.0 (1.6) | 2.9 (1.3) |
| **Blood pressure and anthropometry** |  |  |  |  |  |  |  |
| SBP (SD), mmHg | 129.0 (19.8) | 126.8 (18.5) | 134.9 (22.7) | 130.4 (22.9) | 120.4 (23.0) | 141.4 (27.0) | 140.9 (23.9) |
| RPG (SD), mmol/L | 5.6 (1.1) | 5.6 (1.1) | 5.8 (1.2) | 5.7 (1.2) | 5.4 (1.3) | 5.7 (1.3) | 6.0 (1.3) |
| BMI (SD), kg/m^2^ | 23.4 (3.2) | 23.6 (3.4) | 24.3 (3.5) | 23.0 (3.5) | 21.0 (3.3) | 23.3 (3.7) | 23.2 (3.4) |
| Waist circumference (SD), cm | 79.4 (9.4) | 80.0 (9.6) | 82.0 (10.0) | 78.5 (10.0) | 72.7 (9.4) | 79.2 (10.4) | 81.5 (9.9) |
| Hip circumference (SD), cm | 90.6 (6.6) | 90.4 (6.6) | 91.8 (7.1) | 89.5 (7.1) | 82.0 (9.4) | 88.9 (10.4) | 90.1 (9.9) |
| Waist-to-hip ratio (SD) | 0.88 (0.07) | 0.87 (0.07) | 0.89 (0.07) | 0.88 (0.07) | 0.81 (0.07) | 0.88 (0.07) | 0.90 (0.07) |
|  | **LCC free** | **FCLD survivor** | **FCMD survivor** | **Dead without LCC** | **Dead with FCLD** | **Dead with FCMD** | **Dead with LCC** |
| **Variable** | (n=345,181) | (n=2039) | (n=84,814) | (n=15,983) | (n=973) | (n=6141) | (n=252) |
| Percent body fat (SD), % | 27.6 (8.1) | 28.0 (8.8) | 29.3 (8.7) | 26.6 (8.8) | 23.6 (8.5) | 27.3 (9.2) | 24.0 (9.2) |
| Height (SD), cm | 158.7 (8.2) | 157.3 (8.5) | 158.7 (8.3) | 158.2 (8.5) | 145.2 (8.8) | 155.5 (8.8) | 159.4 (9.0) |
| **Number of high-risk lifestyle factors, %** |  |  |  |  |  |  |  |
| 0 | 9.9 | 7.2 | 5 | 3.9 | 4.2 | 2.6 | 3.8 |
| 1 | 43.4 | 36.9 | 35.7 | 33.8 | 34.4 | 33.3 | 28.4 |
| 2 | 37.8 | 41.5 | 47.3 | 48.3 | 47.8 | 51.7 | 45.5 |
| 3 to 4 | 7.9 | 12.2 | 10.5 | 12.8 | 12.2 | 11.6 | 20.9 |

Abbreviations: BMI=body mass index, CLM=cardiometabolic-liver multimorbidity, FCLD=first chronic liver disease, FCMD=first cardiometabolic disease, MET=metabolic equivalent of task, RPG=random plasma glucose, SBP=systolic blood pressure, TIA=transient ischemic attack.

Results were standardized by age, sex, and region (where appropriate). Values are means unless otherwise stated.

*P*-values of baseline characteristics between participants by numbers of metabolic risk factors: all <0.05.

## Supplementary Table 3. Hazard ratios by number of healthy lifestyle factors by CLD subtypes

|  |  | **HR (95% CI)** | | | | |
| --- | --- | --- | --- | --- | --- | --- |
|  | **No. events** | **0-1** | **2** | **3** | **4** | **per 1 score** |
| **Baseline → FCLD** |  |  |  |  |  |  |
| Baseline → NAFLD | 743 | Reference | 1.16 (0.83, 1.62) | 1.77 (1.27, 2.46) | 3.08 (2.16, 4.38) | 1.54 (1.41, 1.69) |
| Baseline → Viral hepatitis | 1638 | Reference | 1.10 (0.88, 1.37) | 1.25 (1.00, 1.56) | 1.67 (1.30, 2.13) | 1.19 (1.12, 1.27) |
| Baseline → Cirrhosis | 1237 | Reference | 0.98 (0.79, 1.23) | 1.12 (0.90, 1.39) | 1.30 (1.00, 1.68) | 1.11 (1.04, 1.20) |
| Baseline → Liver cancer | 1999 | Reference | 1.26 (1.00, 1.59) | 1.60 (1.27, 2.02) | 2.33 (1.82, 2.98) | 1.33 (1.25, 1.41) |
| **FCLD → LCC** |  |  |  |  |  |  |
| NAFLD → LCC | 182 | Reference | 1.35 (0.64, 2.83) | 1.25 (0.60, 2.60) | 1.34 (0.62, 2.90) | 1.03 (0.86, 1.23) |
| Viral hepatitis → LCC | 178 | Reference | 0.84 (0.42, 1.65) | 1.04 (0.53, 2.03) | 1.54 (0.75, 3.15) | 1.25 (1.03, 1.52) |
| Cirrhosis → LCC | 135 | Reference | 1.30 (0.55, 3.05) | 1.58 (0.68, 3.68) | 2.73 (1.11, 6.75) | 1.38 (1.10, 1.73) |
| Liver cancer → LCC | 86 | Reference | 0.62 (0.18, 2.20) | 1.19 (0.36, 3.96) | 1.26 (0.36, 4.45) | 1.34 (1.00, 1.79) |
| **Baseline → Death** | 15983 | Reference | 1.10 (1.02, 1.20) | 1.31 (1.20, 1.42) | 1.65 (1.51, 1.80) | 1.20 (1.17, 1.23) |
| **FCLD → Death** |  |  |  |  |  |  |
| NAFLD → Death | 25 | Reference | --- | --- | --- | 1.78 (1.06, 3.01) |
| Viral hepatitis → Death | 810 | Reference | 1.36 (0.82, 2.26) | 1.50 (0.91, 2.49) | 2.41 (1.43, 4.09) | 1.30 (1.14, 1.47) |
| Cirrhosis → Death | 391 | Reference | 0.87 (0.56, 1.35) | 1.15 (0.75, 1.77) | 1.50 (0.93, 2.44) | 1.24 (1.08, 1.41) |
| Liver cancer → Death | 1659 | Reference | 1.10 (0.84, 1.43) | 1.08 (0.83, 1.40) | 1.21 (0.91, 1.60) | 1.04 (0.98, 1.11) |
| **LCC → Death** | 645 | Reference | 0.81 (0.48, 1.36) | 0.82 (0.49, 1.36) | 1.21 (0.72, 2.04) | 1.17 (1.06, 1.31) |

Abbreviations: FCLD, first chronic liver disease; FCMD, first cardiometabolic disease; LCC, liver-cardiometabolic comorbidity.

The model was adjusted for age at baseline, sex, study area, education, BMI, HBsAg, and self-rated health, with additional adjustment for the other lifestyle factors.

##

## Supplementary Table 4. Sensitivity analyses

|  | **HR (95% CI)** |  |  |  |
| --- | --- | --- | --- | --- |
|  | **Overall** | **Sensitivity analysis** | | |
|  |  | **1** | **2** | **3** |
| **Per 1-score** |  |  |  |  |
| Baseline → FCLD | 1.30 (1.25, 1.35) | 1.30 (1.25, 1.35) | 1.30 (1.25, 1.35) | 1.30 (1.25, 1.35) |
| FCLD → LCC | 1.21 (1.09, 1.34) | 1.21 (1.09, 1.34) | 1.22 (1.10, 1.35) | 1.22 (1.10, 1.35) |
| Baseline → death | 1.20 (1.17, 1.23) | 1.20 (1.17, 1.23) | 1.20 (1.17, 1.23) | 1.20 (1.17, 1.23) |
| FCLD → death | 1.15 (1.09, 1.22) | 1.15 (1.09, 1.22) | 1.15 (1.09, 1.22) | 1.15 (1.09, 1.22) |
| LCC → death | 1.17 (1.06, 1.31) | 1.17 (1.06, 1.31) | 1.17 (1.06, 1.31) | 1.17 (1.06, 1.31) |
| **Smoking** |  |  |  |  |
| Baseline → FCLD | 1.51 (1.42, 1.61) | 1.51 (1.42, 1.61) | 1.52 (1.42, 1.61) | 1.52 (1.43, 1.62) |
| FCLD → LCC | 1.08 (0.89, 1.30) | 1.08 (0.89, 1.30) | 1.09 (0.91, 1.31) | 1.11 (0.92, 1.34) |
| Baseline → death | 1.68 (1.62, 1.74) | 1.68 (1.62, 1.74) | 1.68 (1.62, 1.74) | 1.68 (1.62, 1.74) |
| FCLD → death | 1.62 (1.48, 1.77) | 1.62 (1.48, 1.77) | 1.62 (1.48, 1.77) | 1.62 (1.48, 1.77) |
| LCC → death | 1.68 (1.42, 1.99) | 1.68 (1.42, 1.99) | 1.68 (1.42, 1.99) | 1.68 (1.42, 1.98) |
| **Alcohol** |  |  |  |  |
| Baseline → FCLD | 1.64 (1.50, 1.79) | 1.64 (1.50, 1.79) | 1.63 (1.50, 1.78) | 1.63 (1.49, 1.77) |
| FCLD → LCC | 1.38 (1.08, 1.76) | 1.38 (1.08, 1.76) | 1.36 (1.06, 1.73) | 1.30 (1.02, 1.67) |
| Baseline → death | 1.30 (1.23, 1.36) | 1.30 (1.23, 1.36) | 1.30 (1.23, 1.36) | 1.30 (1.23, 1.36) |
| FCLD → death | 1.03 (0.91, 1.16) | 1.03 (0.91, 1.16) | 1.03 (0.91, 1.16) | 1.03 (0.91, 1.16) |
| LCC → death | 1.20 (0.96, 1.51) | 1.20 (0.96, 1.51) | 1.20 (0.96, 1.51) | 1.20 (0.96, 1.51) |
| **Physical inactivity** |  |  |  |  |
| Baseline → FCLD | 0.95 (0.89, 1.02) | 0.95 (0.89, 1.02) | 0.96 (0.89, 1.03) | 0.96 (0.89, 1.03) |
| FCLD → LCC | 1.15 (0.93, 1.43) | 1.15 (0.93, 1.43) | 1.19 (0.96, 1.48) | 1.20 (0.96, 1.49) |
| Baseline → death | 1.07 (1.02, 1.12) | 1.07 (1.02, 1.12) | 1.07 (1.02, 1.12) | 1.07 (1.02, 1.12) |
| FCLD → death | 1.27 (1.13, 1.42) | 1.27 (1.13, 1.42) | 1.27 (1.13, 1.42) | 1.27 (1.13, 1.42) |
| LCC → death | 1.18 (0.91, 1.52) | 1.18 (0.91, 1.52) | 1.18 (0.91, 1.52) | 1.18 (0.91, 1.52) |
| **Central adiposity** |  |  |  |  |
| Baseline → FCLD | 1.09 (1.02, 1.16) | 1.09 (1.02, 1.16) | 1.09 (1.02, 1.16) | 1.09 (1.02, 1.16) |
| FCLD → LCC | 1.32 (1.11, 1.57) | 1.32 (1.11, 1.57) | 1.31 (1.10, 1.55) | 1.31 (1.10, 1.55) |
| Baseline → death | 0.73 (0.70, 0.76) | 0.73 (0.70, 0.76) | 0.73 (0.70, 0.76) | 0.73 (0.70, 0.76) |
| FCLD → death | 0.64 (0.58, 0.71) | 0.64 (0.58, 0.71) | 0.64 (0.58, 0.71) | 0.64 (0.58, 0.71) |
| LCC → death | 0.72 (0.61, 0.84) | 0.72 (0.61, 0.84) | 0.72 (0.61, 0.84) | 0.72 (0.61, 0.84) |

Sensitivity analyses 1-3: calculating the entering date of the prior state using different time intervals (1, 3, and 5 days) for participants who entered different states on the same day, respectively.

Sensitivity analysis 4: excluding participants who entered different states on the same date.

Sensitivity analysis 5: additionally adjusting for hypertension, usage of blood pressure medicine, and statin at baseline.

Sensitivity analysis 6: including participants who had previously diagnosed CLD, CHD, stroke, or diabetes, and assigning them to FCLD or CLM state according to their disease status at baseline.

Sensitivity analysis 7: excluding the events occurring in the first 2 years of follow-up.

Sensitivity analysis 8: calculating the entering date of the theoretically prior state as half of the entering date of the latter.

**Supplementary Table 4. Continued**

|  | **HR (95% CI)** |  |  |  |
| --- | --- | --- | --- | --- |
|  | **Sensitivity analysis** | | | |
|  | **4** | **5** | **6** | **7** |
| **Per 1-score** |  |  |  |  |
| Baseline → FCLD | 1.31 (1.26, 1.37) | 1.31 (1.26, 1.37) | 1.30 (1.25, 1.35) | 1.32 (1.27, 1.38) |
| FCLD → LCC | 1.21 (1.09, 1.34) | 1.21 (1.09, 1.34) | 1.22 (1.10, 1.35) | 1.24 (1.09, 1.40) |
| Baseline → death | 1.19 (1.17, 1.22) | 1.19 (1.17, 1.22) | 1.20 (1.17, 1.23) | 1.19 (1.16, 1.22) |
| FCLD → death | 1.22 (1.14, 1.31) | 1.22 (1.14, 1.31) | 1.15 (1.09, 1.22) | 1.08 (1.02, 1.15) |
| LCC → death | 1.17 (1.05, 1.30) | 1.17 (1.05, 1.30) | 1.17 (1.06, 1.31) | 1.13 (1.01, 1.28) |
| **Smoking** |  |  |  |  |
| Baseline → FCLD | 1.52 (1.41, 1.63) | 1.52 (1.41, 1.63) | 1.52 (1.43, 1.62) | 1.55 (1.45, 1.66) |
| FCLD → LCC | 1.14 (0.94, 1.37) | 1.14 (0.94, 1.37) | 1.11 (0.92, 1.34) | 1.21 (0.97, 1.51) |
| Baseline → death | 1.74 (1.68, 1.80) | 1.74 (1.68, 1.80) | 1.68 (1.62, 1.74) | 1.67 (1.61, 1.74) |
| FCLD → death | 1.78 (1.58, 2.01) | 1.78 (1.58, 2.01) | 1.62 (1.48, 1.77) | 1.56 (1.41, 1.72) |
| LCC → death | 1.73 (1.46, 2.04) | 1.73 (1.46, 2.04) | 1.68 (1.42, 1.98) | 1.60 (1.32, 1.94) |
| **Alcohol** |  |  |  |  |
| Baseline → FCLD | 1.67 (1.51, 1.83) | 1.67 (1.51, 1.83) | 1.63 (1.49, 1.77) | 1.66 (1.51, 1.83) |
| FCLD → LCC | 1.29 (1.01, 1.65) | 1.29 (1.01, 1.65) | 1.30 (1.02, 1.67) | 1.26 (0.94, 1.68) |
| Baseline → death | 1.27 (1.20, 1.33) | 1.27 (1.20, 1.33) | 1.30 (1.23, 1.36) | 1.34 (1.27, 1.42) |
| FCLD → death | 1.16 (1.00, 1.35) | 1.16 (1.00, 1.35) | 1.03 (0.91, 1.16) | 0.99 (0.87, 1.14) |
| LCC → death | 1.21 (0.96, 1.52) | 1.21 (0.96, 1.52) | 1.20 (0.96, 1.51) | 1.14 (0.88, 1.47) |
| **Physical inactivity** |  |  |  |  |
| Baseline → FCLD | 0.91 (0.84, 0.98) | 0.91 (0.84, 0.98) | 0.96 (0.89, 1.03) | 1.02 (0.94, 1.11) |
| FCLD → LCC | 1.20 (0.97, 1.50) | 1.20 (0.97, 1.50) | 1.20 (0.96, 1.49) | 1.34 (1.01, 1.78) |
| Baseline → death | 1.08 (1.04, 1.14) | 1.08 (1.04, 1.14) | 1.07 (1.02, 1.12) | 1.01 (0.96, 1.06) |
| FCLD → death | 1.11 (0.97, 1.29) | 1.11 (0.97, 1.29) | 1.27 (1.13, 1.42) | 1.05 (0.92, 1.19) |
| LCC → death | 1.19 (0.92, 1.53) | 1.19 (0.92, 1.53) | 1.18 (0.91, 1.52) | 1.04 (0.78, 1.39) |
| **Central adiposity** |  |  |  |  |
| Baseline → FCLD | 1.17 (1.09, 1.25) | 1.17 (1.09, 1.25) | 1.09 (1.02, 1.16) | 1.05 (0.98, 1.12) |
| FCLD → LCC | 1.24 (1.05, 1.47) | 1.24 (1.05, 1.47) | 1.31 (1.10, 1.55) | 1.25 (1.02, 1.53) |
| Baseline → death | 0.70 (0.67, 0.72) | 0.70 (0.67, 0.72) | 0.73 (0.70, 0.76) | 0.73 (0.70, 0.76) |
| FCLD → death | 0.68 (0.60, 0.78) | 0.68 (0.60, 0.78) | 0.64 (0.58, 0.71) | 0.64 (0.58, 0.72) |
| LCC → death | 0.68 (0.58, 0.80) | 0.68 (0.58, 0.80) | 0.72 (0.61, 0.84) | 0.76 (0.63, 0.91) |

Sensitivity analyses 1-3: calculating the entering date of the prior state using different time intervals (1, 3, and 5 days) for participants who entered different states on the same day, respectively.

Sensitivity analysis 4: excluding participants who entered different states on the same date.

Sensitivity analysis 5: additionally adjusting for hypertension, usage of blood pressure medicine, and statin at baseline.

Sensitivity analysis 6: including participants who had previously diagnosed CLD, CHD, stroke, or diabetes, and assigning them to FCLD or CLM state according to their disease status at baseline.

Sensitivity analysis 7: excluding the events occurring in the first 2 years of follow-up.

Sensitivity analysis 8: calculating the entering date of the theoretically prior state as half of the entering date of the latter.

**Supplementary Table 4. Continued**

|  | **HR (95% CI)** |
| --- | --- |
|  | **Sensitivity analysis** |
|  | **8** |
| **Per 1-score** |  |
| Baseline → FCLD | 1.46 (1.41, 1.51) |
| FCLD → LCC | 1.23 (1.13, 1.33) |
| Baseline → death | 1.17 (1.15, 1.20) |
| FCLD → death | 0.99 (0.94, 1.05) |
| LCC → death | 1.12 (1.01, 1.24) |
| **Smoking** |  |
| Baseline → FCLD | 1.69 (1.59, 1.79) |
| FCLD → LCC | 1.01 (0.88, 1.16) |
| Baseline → death | 1.67 (1.61, 1.73) |
| FCLD → death | 1.36 (1.24, 1.48) |
| LCC → death | 1.36 (1.16, 1.61) |
| **Alcohol** |  |
| Baseline → FCLD | 1.54 (1.42, 1.68) |
| FCLD → LCC | 1.13 (0.93, 1.38) |
| Baseline → death | 1.31 (1.25, 1.38) |
| FCLD → death | 1.19 (1.06, 1.34) |
| LCC → death | 1.36 (1.09, 1.70) |
| **Physical inactivity** |  |
| Baseline → FCLD | 1.27 (1.18, 1.36) |
| FCLD → LCC | 1.39 (1.14, 1.71) |
| Baseline → death | 1.01 (0.96, 1.05) |
| FCLD → death | 0.83 (0.74, 0.93) |
| LCC → death | 0.95 (0.74, 1.23) |
| **Central adiposity** |  |
| Baseline → FCLD | 1.19 (1.12, 1.26) |
| FCLD → LCC | 1.56 (1.37, 1.76) |
| Baseline → death | 0.70 (0.68, 0.73) |
| FCLD → death | 0.59 (0.54, 0.65) |
| LCC → death | 0.85 (0.72, 0.99) |

Sensitivity analyses 1-3: calculating the entering date of the prior state using different time intervals (1, 3, and 5 days) for participants who entered different states on the same day, respectively.

Sensitivity analysis 4: excluding participants who entered different states on the same date.

Sensitivity analysis 5: additionally adjusting for hypertension, usage of blood pressure medicine, and statin at baseline.

Sensitivity analysis 6: including participants who had previously diagnosed CLD, CHD, stroke, or diabetes, and assigning them to FCLD or CLM state according to their disease status at baseline.

Sensitivity analysis 7: excluding the events occurring in the first 2 years of follow-up.

Sensitivity analysis 8: calculating the entering date of the theoretically prior state as half of the entering date of the latter.

## Supplementary Table 5. Hazard ratios by number of healthy lifestyle factors by CMD subtypes

|  |  | **HR (95% CI)** | | | | |
| --- | --- | --- | --- | --- | --- | --- |
|  | **No. events** | **0-1** | **2** | **3** | **4** | **per 1 score** |
| **FCLD → LCC** |  |  |  |  |  |  |
| CHD → LCC | 182 | Reference | 0.79 (0.41, 1.52) | 1.02 (0.53, 1.91) | 0.90 (0.45, 1.81) | 1.06 (0.88, 1.27) |
| Stroke → LCC | 318 | Reference | 0.93 (0.53, 1.65) | 1.34 (0.77, 2.33) | 1.75 (0.98, 3.12) | 1.32 (1.14, 1.52) |
| Diabetes → LCC | 152 | Reference | 1.59 (0.68, 3.73) | 1.78 (0.77, 4.11) | 2.08 (0.86, 5.00) | 1.19 (0.98, 1.44) |

Abbreviations: FCLD, first chronic liver disease; FCMD, first cardiometabolic disease; LCC, liver-cardiometabolic comorbidity.

The model was adjusted for age at baseline, sex, study area, education, BMI, HBsAg, and self-rated health, with additional adjustment for the other lifestyle factors.
